# The C terminus of NS5A domain II is a key determinant of hepatitis C virus genome replication, but is not required for virion assembly and release

**DOI:** 10.1099/vir.0.050633-0

**Published:** 2013-05

**Authors:** Douglas Ross-Thriepland, Yutaka Amako, Mark Harris

**Affiliations:** School of Molecular and Cellular Biology, Faculty of Biological Sciences and Astbury Centre for Structural Molecular Biology, University of Leeds, Leeds, LS2 9JT, UK

## Abstract

The NS5A protein of hepatitis C virus (HCV) plays roles in both virus genome replication and the assembly of infectious virus particles. NS5A comprises three domains, separated by low-complexity sequences. Whilst the function of domain I appears to be predominantly involved with genome replication, the roles of domains II and III are less well defined. It has been reported previously that a deletion spanning the majority of domain II but retaining the C-terminal 35 residues had no effect on virus production; however, deletion of the entire domain II eliminated genome replication, pointing to a key role for the C terminus of this domain. Recent work has also highlighted this region as the potential binding site of the host factor cyclophilin A (CypA). To define this requirement for replication in more detail, and to investigate the involvement of CypA, we conducted a mutagenic study of the C-terminal 30 residues of domain II within the context of both the infectious JFH-1 virus and a JFH-1-derived subgenomic replicon. We showed that 12 of these residues were absolutely required for virus genome replication, whilst mutations of the remainder either had no phenotype or exhibited a partial reduction in genome replication. There was an absolute correlation between the datasets for virus and subgenomic replicon, indicating that this region is involved solely in the process of genome replication. Comparison of our data with a previously published analysis of the same region in genotype 1b revealed some important differences between the two genotypes of HCV.

## Introduction

Hepatitis C virus (HCV) currently infects ~2–3 % of the world’s population, of whom around 85 % will progress to chronic infection where the major outcome is liver cirrhosis and hepatocellular carcinoma (Shepard *et al.*, 2005). Current treatment is a combination of pegylated interferon and ribavirin, but recently the first direct-acting antivirals targeting the NS3 protease were licensed and numerous other drugs are in late-stage clinical trials (Yang *et al.*, 2011). Of particular interest are compounds targeted to the NS5A protein such as BMS-790052, which show extraordinarily high potency (Gao *et al.*, 2010). However, toxicity and the development of drug resistance are associated with all treatments, highlighting the need for further research.

HCV has a 9.6 kb positive-sense ssRNA genome coding for a ~3000 aa polyprotein that is cleaved co- and post-translationally by both host and viral proteases to produce ten mature viral proteins. The structural proteins – core protein, E1 and E2 – are involved in the structure and formation of virus particles, p7 is a viroporin that may or may not be a virion component but has roles in virus assembly and release (Griffin 2010), and non-structural protein 2 (NS2) contains an autoprotease activity that cleaves it from NS3 but also has a poorly defined role in virion morphogenesis (Jirasko *et al.*, 2008; Ma *et al.*, 2011). The remaining non-structural proteins – NS3, NS4A, NS4B, NS5A and NS5B – are responsible for replication of the viral genome and remodelling of the host-cell architecture and physiology in favour of HCV replication and persistence (reviewed by Moradpour *et al.*, 2007). Recently, a full-length clone of a genotype 2a isolate, termed JFH-1, was shown to be able to undergo the complete virus life cycle in cell culture without the need for adaptive mutations, allowing stages of virion assembly and release to be investigated for the first time (Wakita *et al.*, 2005).

**Fig. 1.**
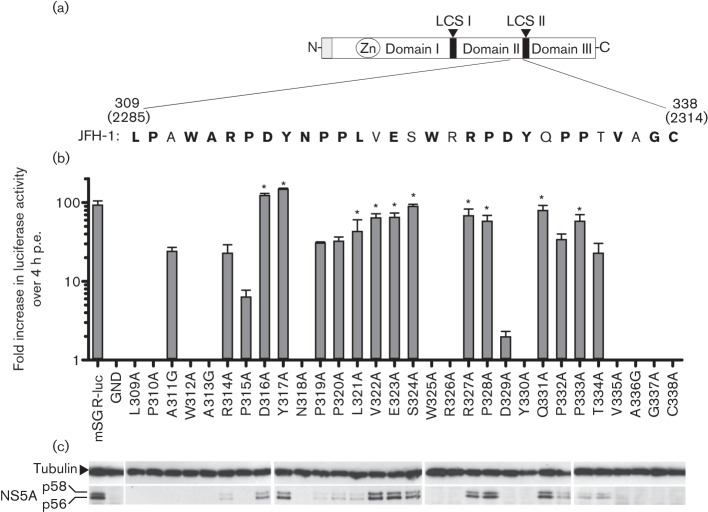
Role of NS5A domain II in replication of the genotype 2a JFH-1 SGR. (a) Sequence of domain II of NS5A targeted for mutagenesis. The organization of the NS5A protein is shown, highlighting the region of domain II that was mutated in this study. Residue numbering is shown in relation to the NS5A sequence of JFH-1 (upper numbers) and the JFH-1 polyprotein sequence (lower numbers). Residues in bold are the consensus sequence conserved in the majority of genotypes (see also Fig. 5 and Fig. S1, available in JGV Online). (b) Huh7 cells were electroporated with mSGR RNA and seeded into either 96-well plates (for the luciferase assay) or six-well plates (for Western blotting). Cells were harvested at 72 h p.e. The luciferase data shown is the mean±sem of more than three experiments and was normalized to luciferase activity at 4 h p.e. Asterisks denote no statistical difference (*P*<0.05) from the WT. (c) Cell lysates were analysed by SDS-PAGE (7.5 % acrylamide) and Western blotting for the indicated proteins.

The NS5A protein comprises three domains linked by two low-complexity sequences (LCSs) (Fig. 1a) that are either serine or proline rich (termed LCS I and II, respectively). Domain I is a highly structured, zinc-binding domain whose three-dimensional structure shows two different dimeric conformations (Love *et al.*, 2009; Tellinghuisen *et al.*, 2005). Domains II and III have been shown to be natively unstructured, but nuclear magnetic resonance (NMR) and circular dichroism have shown that both these domains have elements of secondary structure throughout (Feuerstein *et al.*, 2012; Hanoulle *et al.*, 2009b; Liang *et al.*, 2007). The protein is anchored to membranes by an N-terminal amphipathic helix and is an essential component of the viral genome replication complex (reviewed by He *et al.*, 2006; Macdonald & Harris 2004), interacting with other non-structural proteins, for example the NS5B polymerase (Shirota *et al.*, 2002), and cellular factors such as human vesicle-associated membrane protein-associated protein A (Evans *et al.*, 2004). NS5A is also a highly phosphorylated protein, existing predominantly in two species: a basally phosphorylated (56 kDa) and a hyperphosphorylated (58 kDa) form. This phosphorylation is widely postulated to act as a molecular switch regulating the different functions of NS5A (Appel *et al.*, 2005).

The NS5A protein has been ascribed a large number of functions in multiple stages of the virus life cycle. As well as perturbing multiple host pathways, it plays critical roles in both virus replication and the assembly of infectious virions. In this regard, NS5A has been shown to possess *in vitro* RNA-binding activity, as the entire protein, or each individual domain, expressed in *Escherichia coli* and purified, have all been shown to bind genome-derived RNA sequences (Foster *et al.*, 2010; Hwang *et al.*, 2010).

Domain II of NS5A is known to be required for replication (Appel *et al.*, 2008), as well as interacting with both host and viral proteins (Evans *et al.*, 2004; Goh *et al.*, 2001; Shirota *et al.*, 2002). Specifically, in the context of JFH-1, a deletion of the C-terminal 35 aa resulted in complete abrogation of replication, whereas, conversely, deleting the preceding N-terminal residues of domain II had no effect on virus replication (Appel *et al.*, 2008). A separate study utilizing a genotype 1b subgenomic replicon (SGR) found that small deletions (8–15 residues) within the C-terminal 56 aa of domain II completely abrogated replication, and further analysis by site-directed mutagenesis identified 23 essential residues within this region (Tellinghuisen *et al.*, 2008b). However, due to the genotype utilized, it was not possible to investigate the role of these residues in the context of the fully infectious virus.

Work has begun to shed light on the potential mechanisms behind this requirement of domain II for replication, most notably involving the host factor cyclophilin A (CypA). CypA is a peptidyl-prolyl isomerase (PPIase) identified as a critical host factor for the successful infection of many viruses, including human immunodeficiency virus, influenza and recently HCV (Fischer *et al.*, 2010). Disruption of its PPIase activity through chemical inhibitors such as cyclosporin (CsA), active-site mutations or small interfering RNA (siRNA) knockdown has been shown to be deleterious to HCV replication. NMR studies also suggest that CypA binds via its active site to the C-terminal region of domain II (Yang *et al.*, 2010), and mutations that confer a loss of CypA dependency cluster within this region (Coelmont *et al.*, 2010). This all points towards the domain II of NS5A forming an interaction with CypA that is critical for replication, and that disruption of this by mutation/deletion in domain II is inhibitory to HCV replication.

In this study, we aimed to fully characterize the residues within this region that are required for either genome replication or the assembly and release of infectious virus. Furthermore, we aimed to establish whether residues essential for replication correspond to the putative binding sites of CypA. To achieve this, we conducted alanine scanning mutagenesis of the C-terminal 30 aa of domain II in the context of the JFH-1 isolate and investigated the phenotype of these mutants within the HCV life cycle. Lastly, we investigated whether mutations in this region disrupted the dependence on CypA for replication by measuring resistance to CypA inhibition.

## Results

### Site-directed mutagenesis of NS5A aa 309–338 identifies numerous residues essential for genome replication

To identify whether residues within domain II are dispensable for the HCV life cycle, we undertook a comprehensive alanine scanning mutagenesis of the C-terminal 30 aa in domain II of NS5A (Fig. 1a). Single residues were mutated to alanine by site-directed mutagenesis (or in the case of alanines in the original sequence, were mutated to glycine) and cloned into a luciferase-based SGR, mSGR-luc-JFH-1 (see Methods), or a JFH-1 infectious clone construct (mJFH-1) (Hughes *et al.*, 2009a). To investigate the effect of these substitutions, *in vitro* transcripts of the panel of mutants, in both the replicon and virus, were electroporated into Huh7 cells and the phenotype determined by luciferase activity (mSGR) or genome quantification by real-time quantitative reverse transcriptase-PCR (qRT-PCR) (mJFH-1).

*In vitro* transcripts of the mSGR-luc-JFH-1 construct were electroporated into Huh7 cells and luciferase activity was measured at 4, 24, 48 and 72 h post-electroporation (p.e.). Replication levels of mutant and WT constructs are shown at 72 h p.e. normalized to the 4 h p.e. signal (Fig. 1b). The 4 h p.e. value was representative of both electroporation efficiency and input translation. As a negative control, the NS5B polymerase inactive mutation (with mutation of the conserved GDD motif to GND) was used (SGR-luc-GND) (Targett-Adams & McLauchlan, 2005).

Twelve mutations (L309A, P310A, W312A, A313G, N318A, W325A, R326A, Y330A, V335A, A336G, G337A and C338A) were shown to completely disrupt the ability of mSGR-luc-JFH-1 to replicate in Huh7 cells, whilst a further eight mutations (A311G, R314A, P315A, P319A, P320A, D329A, P332A and T334A) resulted in a significant reduction (*P*>0.05) compared with WT replication. Ten mutations (D316A, Y317A, L321A, V322A, E323A, S324A, R327A, P328A, Q331A and P333A) showed no statistically significant difference (*P*<0.05) from WT mSGR-luc-JFH-1 replication.

To further support the luciferase data, total protein at 72 h p.e. was analysed by Western blotting for both NS5A and tubulin. Fig. 1(c) shows that, in the case of 15 of the 18 replicating mutants, both phosphorylated forms of NS5A could be detected at levels broadly correlating with the observed level of replication measured by the luciferase assay. However, for mutants A311G, P315A and D329A, the NS5A protein could not be detected, even at higher exposures of the Western blot (data not shown). For P315A and D329A, the absence of NS5A was expected, due to the very low levels of replication observed, but A311G replicated to levels comparable to R314A and T334A, both of which had detectable levels of NS5A in cell lysates. This issue is addressed later in Fig. 3.

**Fig. 2.**
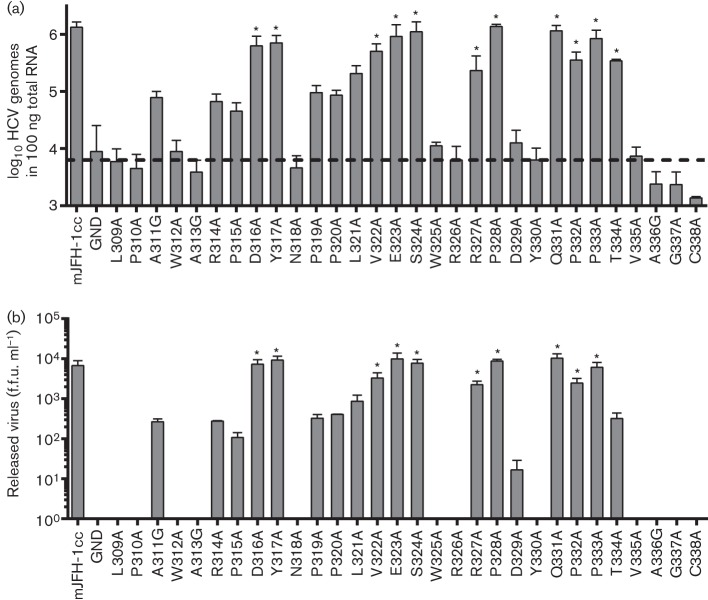
Effect of NS5A domain II mutations on JFH-1 virus replication and assembly/release. (a) Huh7 cells were electroporated with full-length mJFH-1 RNA, incubated for 72 h before being passaged 1 : 5 and incubated for a further 72 h. At 144 h p.e., the cells were harvested in TRIzol, total RNA was extracted and qRT-PCR was conducted on 100 ng total cellular RNA using 5′-UTR TaqMan primers. (b) Huh7 cells were electroporated with mJFH-1 RNA and incubated for 72 h before being passaged 1 : 5 and incubated for a further 72 h. At 144 h p.e., the supernatants were harvested and used to determine the titre of release virus by focus-forming assay. Asterisks denote no statistical difference (*P*<0.05) from the WT. f.f.u., Focus-forming units.

To examine whether the mSGR-luc-JFH-1 replication phenotypes were also observed in the context of the complete virus life cycle, the panel of substitutions was cloned into the full-length mJFH-1 infectious clone. *In vitro* transcripts were electroporated into Huh7 cells as described in Methods and incubated for a total of 144 h, with a 1 : 5 passage at 72 h p.e. At 144 h p.e., total RNA was extracted from the cells and the HCV genomes quantified using qRT-PCR. A 144 h incubation time was required in order to allow the degradation of input RNA in non-replicating mutants. However, despite this, the negative-control JFH-1 GND mutant still maintained levels of HCV genomes (or fragments thereof) at significant levels at 144 h p.e. This background level of HCV RNA at 144 h p.e. suggested that the 5′-UTR (the target of the qRT-PCR primers) is highly resistant to degradation by cellular RNases, possible due to the highly structured nature of the 5′-UTR as well as the stabilizing effect of the microRNA miR-122 (Shimakami *et al.*, 2012). There was, however, a sufficient window between the WT and the GND negative control to identify a genome replication phenotype for the panel of mutations. The quantification of HCV genomes carrying domain II mutations at 144 h p.e. is shown in Fig. 2(a). The replication phenotype of the mutant panel in the context of full-length virus correlated well with the data observed in the mSGR-luc-JFH-1 system.

### Domain II plays no significant role in the assembly or release of infectious virions

We and others (Tellinghuisen *et al.*, 2008a) have previously identified residues in LCS II and domain III of NS5A that have no phenotype in genome replication but play roles in the later stages of the virus life cycle. For example, the P342A mutation has no effect on genome replication but reduces infectious virus titres by 1 log (Hughes *et al.*, 2009b). We considered that this might also be the case for those residues within domain II identified in Figs 1 and 2 with no apparent role in genome replication. To investigate this, RNA transcripts of full-length mJFH-1 virus containing domain II mutations were electroporated into Huh7 cells, which were passaged 1 : 5 at 72 h p.e., prior to determination of released virus titre at 144 h p.e. by focus-forming assay. As previously, the negative control was the JFH-1 GND construct.

Analysis of the effect of domain II mutations on the release of infectious virus (Fig. 2b) showed that there was a broad correlation with the genome replication data. Specifically, those mutants with a WT genome replication phenotype also produced WT levels of infectious virus, and those that were reduced significantly from WT for genome replication also showed a significant reduction in infectious virus. We concluded that the reduction in virus titre observed for domain II mutations stemmed from impairment to replication and not as a result of specific disruption to the virus assembly/release pathways. These data provide further support for the conclusion that the C terminus of domain II plays a pivotal role in HCV genome replication but has no role in the assembly or release of infectious virus particles.

### Mutations that eliminate RNA replication do not disrupt polyprotein processing

**Fig. 3.**
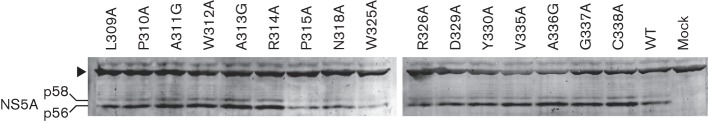
Expression of non-replicating NS5A domain II mutants. Huh7 cells seeded in six-well plates were transfected with pCMV10-NS3-5B DNA and harvested at 48 h for Western blot analysis. Lysates were separated by SDS-PAGE (7.5 % acrylamide) and probed with anti-NS5 antibodies and a sheep polyclonal anti-NS5A serum, which reacts with a cellular protein (arrowhead), acting as a loading control.

In the case of non-replicating mutations, it was important to establish that this elimination of replication resulted from a loss or disruption of a specific function of NS5A, rather than disruption of polyprotein translation or proteolytic processing. To demonstrate this, the 12 non-replicating mutants, together with the three mutants for which NS5A expression could not be observed (A311G, P315A and D329A; Fig. 1b) and R314A as a representative mutant with an intermediate phenotype, were cloned into an expression construct in which the NS3–5B coding sequences of JFH-1 were expressed from a human cytomegalovirus (CMV) promoter (pCMV-10-NS3-5B) (Jones *et al.*, 2009), thus allowing replication-independent expression of the replicase proteins. These expression constructs were transfected into Huh7 cells and analysed for protein expression by Western blotting (Fig. 3). All of the mutants expressed both phosphorylated forms of NS5A (the basal p56 and hyperphosphoylated p58), confirming that the lethal replication phenotype of these mutations was a direct result of a loss of NS5A function and was not due to global effects on NS5A structure or inhibition of polyprotein processing. Notably, mutants P315A and W325A exhibited lower levels of expression in comparison with the WT. This may suggest that these two mutants affected the stability of the protein; however, pulse–chase analysis would be required to confirm this hypothesis. Intriguingly, A311G, which was undetectable in the context of a replicating SGR, could be detected at WT levels in this assay. This suggests that additional factors may influence the stability of NS5A when incorporated into active RNA replication complexes.

### Mutation of residues within two potential CypA-binding motifs has a limited effect on the sensitivity of replication to CypA inhibition

The function of CypA has been shown to be critical for the successful replication of HCV. CypA-independent mutations have been identified by both CsA treatment and siRNA knockdown of CypA (Coelmont *et al.*, 2010; Yang *et al.*, 2010). Both techniques identified key resistant mutations that cluster in this C-terminal region of domain II, specifically R314W, D316E, Y317N (aa 318, 320 and 321 in the HCV Con1 isolate, respectively). When these mutations were inserted into the replicon (either individually or in combination), there was a consistent (and additive) increase in the 50 % effective concentration (EC_50_) of drugs that inhibit CypA.

**Fig. 4.**
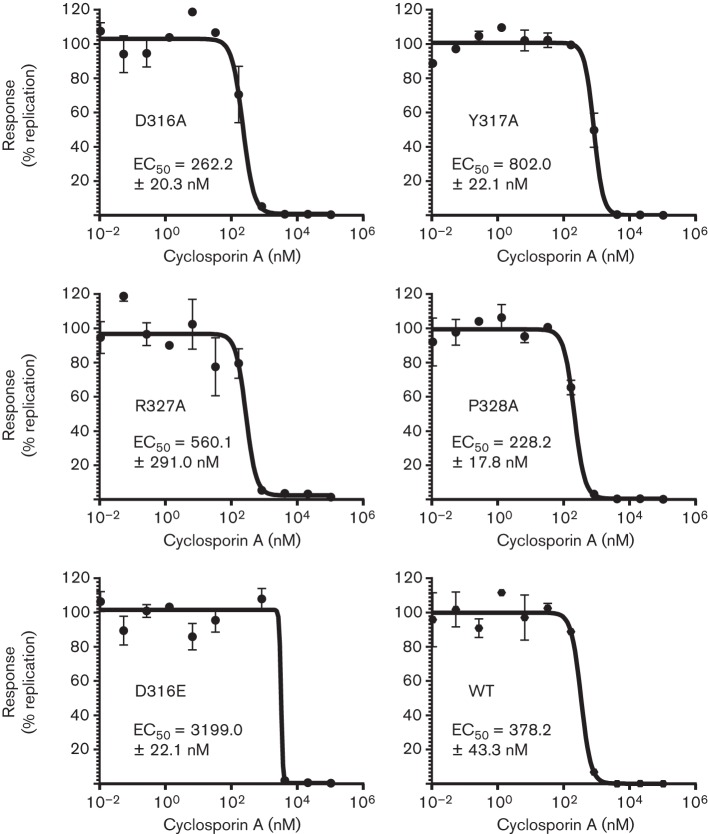
Effect of domain II mutations on CsA EC_50_. Huh7 cells were electroporated with mSGR RNA, seeded into 96-well plates and treated at 4 h p.e. with serial dilutions of CsA. At 48 h p.e., the cells were harvested and the luciferase activity analysed. The resulting luciferase activity was normalized to the maximum signal and the data processed using Prism (GraphPad) using a standard EC_50_ model.

These resistant residues are located within a motif (RPDY) that is present twice within this region of domain II (R314–Y317 and R327–Y330; Fig. 1a). As mutations in residues in both of these motifs showed a reduction in genome replication (R314, P315, D329 and Y330), we considered that this might be explained by a loss of CypA dependence. To test this, the EC_50_ of CsA inhibition for mutations of these residues was determined. Huh7 cells were electroporated with *in vitro* transcripts of mutant and WT mSGR-luc-JFH-1, treated at 4 h p.e. with CsA (0.01–100 µM, 0.5 % DMSO final concentration) and luciferase activity was determined at 48 h p.e. (Fig. 4). In parallel, (3-(4,5-dimethylthiazol-2-yl)-2,5-diphenyltetrazolium bromide (MTT) assays were conducted to determine the 50 % cytotoxic concentration (CC_50_) to confirm that any reduction in luciferase activity was not a result of cytotoxicity. CsA was not toxic at any of the concentrations used (data not shown). As experimental controls, the CsA-resistant mutation D316E was used to demonstrate an increase in EC_50_, and the NS5A inhibitor BMS-790052 was used as a negative control where no shift in EC_50_ was expected (Fridell *et al.*, 2010).

In the case of three mutants (R314A, P315A in the first motif and D329A in the second), the impairment of replication resulting from alanine substitution was too great to allow an accurate CsA EC_50_ to be determined; however, the sensitivity of these mutants to CsA inhibition was broadly comparable to that of WT (data not shown). For the remaining four mutations (D316A, Y317A, R327A and P328A), an accurate EC_50_ (Fig. 4) could be determined. Mutations D316A and P328A resulted in an ~50 % increase in sensitivity to CsA treatment (decreased EC_50_) when compared with the WT. Conversely, the Y317A and R327A mutations resulted in a modest increase in CsA EC_50_, and, although it was not as dramatic as that of the major resistance mutation D316E, which showed an expected 8.5-fold increase in EC_50_, it was still significant. As expected, BMS-790052 was equally effective against both WT and D316E (EC_50_ of 17.4±5.0 and 24.2±2.1 pM, respectively; data not shown). These data showed that mutations within this region of domain II are able to modulate the sensitivity of replication to CypA inhibition by CsA.

## Discussion

In this study, we utilized the HCV genotype 2a isolate JFH-1 to investigate the role of the C-terminal 30 residues of NS5A domain II within the virus life cycle. We identified 12 residues within this region that were necessary for viral genome replication, and substitutions of these amino acids to alanine (or to glycine in the case of alanine residues within NS5A) eliminated genome replication in both full-length virus and SGRs. A further eight mutations resulted in a significant inhibition of replication, implying that, although these residues are not essential, they do contribute to genome replication. There was, however, no residue within this region that, when mutated, resulted in a reduction in released virus titre without a corresponding reduction in virus replication, implying that this region is exclusively involved in genome replication. These data reinforce the conclusion that the primary role of domain II is in genome replication, and that those residues not required for replication play no role in the release of infectious virions. However, we cannot rule out the possibility that residues essential for replication are also required for virus assembly and/or release, although such a duality in function is unlikely and is technically challenging to address experimentally.

### Genotype-specific requirements within domain II for RNA replication

Two previous studies have assessed the requirements for genome replication of the corresponding region in the genotype 1b SGR (Shimakami *et al.*, 2004; Tellinghuisen *et al.*, 2008b), but as the genotype 1b isolates were not able to undergo infection in cell culture, investigation of the later stages of the virus life cycle was precluded. The data generated in the study presented here allowed us to compare the residues required for genome replication between the two genotypes.

**Fig. 5.**
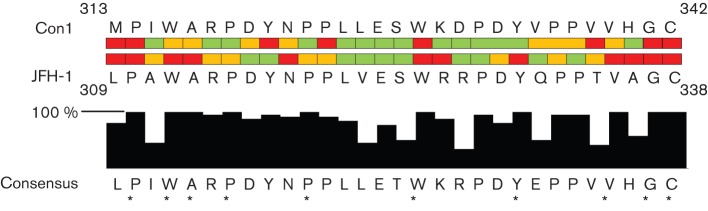
Requirement of residues within the C-terminal region of domain II for genome replication in the JFH-1 and Con1 isolates. The effect of alanine mutation on RNA replication was screened by a colony-forming assay (Con1) (Tellinghuisen *et al.*, 2008b) or by luciferase reporter/qRT-PCR (JFH-1; this study). Red, non-replicating; orange, partial and statistically significant impairment of replication; green, no effect on replication. The lower part of the figure shows the consensus across this region based on a subset of isolates representing each genotype (Simmonds *et al.*, 2005). A full alignment of these sequences is shown in Fig. S1. Filled bars indicate the percentage conservation at each residue, whilst asterisks denote residues that are 100 % conserved.

As shown in Fig. 5, this region exhibited a high level of sequence conservation across the genotypes. Ten of the 30 residues were absolutely conserved, and a further ten showed a very high level of sequence conservation (≥90 %; Fig. 5). One prediction would be that those residues that are absolutely conserved would also play critical roles; however, this was not entirely the case. Although P310, W325, G337 and C338 are essential for genome replication in genotypes 1b and 2a, the data for other conserved residues is less clear cut. For example, in this study, mutations of residues W312, A313, Y330 and V336 were lethal, but in genotype 1b the corresponding mutations have either a null or partial phenotype.

Focusing on the differences between Con1 and JFH-1, 22 residues were conserved between the two genotypes, yet in only a minority of these residues did the requirements match (Fig. 5). In fact, only five residues appeared to be dispensable for both genotypes. Overall within this region, 77 % of residues in the JFH-1 isolate were required for genome replication compared with only 50 % in the Con1 isolate, indicating that this region appears to have greater importance for genome replication in the JFH-1 isolate.

How might these differences be explained? One possibility might be in the context of protein–protein interactions, as this region of NS5A has been reported to interact with the NS5B polymerase (Shimakami *et al.*, 2004) and the cellular factor CypA (Hanoulle *et al.*, 2009a; Yang *et al.*, 2010). Furthermore, a recent NMR study predicted that this region is the least disordered in domain II with no propensity to form a helical structure; however, residues within this region underwent chemical shift changes upon incubation with the Bin1 SH3 domain (Feuerstein *et al.*, 2012), indicating that they were directly involved in the interaction. It is therefore possible that these interactions might be subtly different between the two genotypes.

### Several residues essential for replication map to CypA interaction sites

As mentioned above, this region of NS5A has been shown to interact with the cellular PPIase CypA. This interaction and the PPIase activity of CypA have been shown to be of critical importance for virus replication, independent of the effects of CypA on the calcineurin pathway (Coelmont *et al.*, 2010). Residues A311–N318, T334, G337 and C338 have been shown by NMR to undergo chemical shifts upon incubation with CypA (Hanoulle *et al.*, 2009a; Yang *et al.*, 2010), suggestive of a direct interaction. In contrast, we (Foster *et al.*, 2011) and others (Chatterji *et al.*, 2010) have shown that D316 and Y317 are not required for CypA binding *in vitro*. Our data presented here are consistent with this latter observation, as, although D316A and Y317A had no apparent phenotype, mutation of the surrounding residues to alanine (or to glycine in the case of A311 and A313) was sufficient either to strongly impair or to completely abolish virus replication. Somewhat paradoxically, D316 and Y317 represent the major determinant for CypA dependence and CsA susceptibility, and a double mutant (D316E, Y317N) exhibited 20-fold resistance to CsA and, unlike WT JFH-1, was able to replicate efficiently in CypA-silenced cells (Yang *et al.*, 2010). Taken together, these data are thus consistent with the previously proposed hypothesis that binding of CypA to the motif PAWARP between residues 310 and 315 is important for HCV genome replication (Hanoulle *et al.*, 2009a).

Intriguingly, this motif overlaps with a longer sequence that is present twice within the region of interest, WxRPDYxPP (where x is any amino acid) and has a high level of conservation across all major genotype isolates (Fig. 5). When comparing both occurrences of this motif in JFH-1, the only consistent pattern that emerged was that W312 and W325, along with the residues C-terminal to these (A313 and R326), are both absolutely required. However, in the Con1 isolate, only the W329A mutation (corresponding to W325A in JFH-1) was lethal, and mutation of W316A (JFH-1 W312A) had only a partial defect. Mutations of the RP residues in the N-terminal motif impaired replication, whereas the same mutations in the C-terminal motif had no effect, and the opposite was true of the DY residues. It would be interesting to analyse the phenotype of combinatorial mutations to determine whether there is any redundancy resulting from the duplication. A further twist to this story is the observation that, in Con1 (Tellinghuisen *et al.*, 2008b), the phenotypes of mutations of the two DY motifs are opposite to the JFH-1 phenotype. D320 and Y321 (corresponding to JFH-1 D316 and Y317) are required for genome replication, whereas D333 and Y334 (JFH-1 D329 and Y330) are not required. These data argue strongly that it is difficult to extrapolate between datasets derived from genotypes 1b and 2a, suggesting that there may well be significant functional differences between the NS5A proteins of these two genotypes. An additional confounding factor is the observation that the phenotype of selected mutants in the genotype 1b replicon is influenced by the combination of culture-adaptive mutations present within the remainder of the replicon. For example, P324A (corresponding to JFH-1 P320A) is lethal in the context of the GIT replicon (mutations E1202G and T1280I in NS3, and K1846T in NS4A) but only displays a partial defect in the NS5A mutant S2201I replicon (Tellinghuisen *et al.*, 2008b).

In conclusion, we have shown that the C-terminal 30 residues of the NS5A domain II have no role in either the assembly or release of infectious virus in the genotype 2a isolate JFH-1, and that the function of this region is restricted to that of the replication of viral genomic RNA. Although this role in genomic replication is consistent with data obtained for genotype 1b, our data highlighted phenotypic variations between genotypes irrespective of the high sequence conservation and caution against extrapolation of datasets between genotypes. A key challenge for the future is to determine the mechanisms underpinning how single amino acid changes can completely eliminate the function of a protein that holds no intrinsic enzymatic function. In particular, it will be intriguing to determine whether any of these changes influence the ability of domain II to interact with either cellular or viral proteins, or indeed viral RNA. Such studies are currently under way in our laboratory.

## Methods

### 

#### Cell culture.

Huh7 cells were cultured in Dulbecco’s modified Eagle’s medium (DMEM; Sigma) supplemented with 10 % FBS, 100 IU penicillin ml^−1^, 100 µg streptomycin ml^−1^ and 1 % non-essential amino acids in a humidified incubator at 37 °C in 5 % CO_2_. For virus propagation, the medium was supplemented with 25 mM HEPES.

#### DNA constructs.

DNA constructs of the full-length pJFH-1 virus (Wakita *et al.*, 2005) or a luciferase reporter SGR (SGR-luc-JFH-1) (Targett-Adams & McLauchlan 2005) were used throughout. Previously, unique restriction sites flanking NS5A, *Bam*HI and *Afe*I, were introduced into both the full-length virus and SGR-luc-JFH-1 constructs (denoted as mJFH-1 and mSGR-luc-JFH-1, respectively) and shown to have no effect on virus genome replication or assembly and release (Hughes *et al.*, 2009a). Mutagenesis of NS5A was performed directly on mSGR-luc-JFH-1 constructs or a LITMUS28i (NEB) subclone of a NS5A containing an *Nsi*I–*Hin*dIII fragment of the JFH-1 cDNA by QuikChange site-directed mutagenesis (Stratagene) and then cloned into either mSGR-luc-JFH-1 or mJFH-1 via the flanking *Bam*HI/*Afe*I sites. The pCMV10-NS3-5B [NS5A(GFP)] vector (Jones *et al.*, 2009) was reverted to WT NS5A by cloning in the *Rsr*II–*Sfi*I fragment from mJFH-1, and the NS5A domain II mutants were then inserted into this WT vector by cloning the *Nsi*I–*Rsr*II fragment containing the mutation from the corresponding mJFH-1 vector. All mutations were verified by sequencing. Plasmid and primer sequences are available on request.

#### DNA transfection.

Huh7 cells were seeded at a concentration of 2×10^5^ cells per well into six-well plates 24 h prior to transfection with 3 µg pCMV-NS3-5B construct using polyethylenimine. At 48 h cells, were washed twice in PBS and lysed in Glasgow lysis buffer [GLB; 1 % Triton X-100, 120 mM KCl, 30 mM NaCl, 5 mM MgCl_2_, 10 % glycerol, 10 mM PIPES/NaOH (pH 7.2)].

#### *In vitro* transcription.

The DNA constructs, mSGR-luc JFH-1 or mJFH-1, were linearized by *Xba*I and overhanging ends were degraded by mungbean nuclease treatment. The DNA was phenol/chloroform extracted and 1 µg linearized DNA was used as template in a 20 µl RiboMAX reaction (Promega). The RNA transcripts were purified by phenol/chloroform extraction and quantified by absorbance at 260 nm. The RNA was also analysed by agarose gel electrophoresis to confirm integrity and quantification.

#### Luciferase-based replication assay.

Huh7 cells were washed twice in DEPC-treated PBS before electroporating 4×10^6^ cells in DEPC-treated PBS with 2 µg RNA at 950 µF and 270 V. The cells were resuspended in complete medium before being seeded into both 96-well plates (*n* = 6) at 3×10^4^ cells per well and six-well plates (*n* = 2) at 3×10^5^ cells per well; both plates were incubated under cell-culture conditions. After 4, 24, 48 and 72 h p.e., cells were harvested by lysis with 30 or 200 µl Passive Lysis Buffer (Promega) for the 96- and six-well plates, respectively. Luciferase activity was determined for the 96-well samples on a BMG Labtech plate reader by automated addition of 50 µl LarI reagent (Promega) and total light emission was monitored over 12 s.

#### SDS-PAGE and Western blotting.

Cells were washed twice with PBS, lysed by resuspension in GLB containing protease and phosphatase inhibitors, and incubated on ice for 15 min. Insoluble material was pelleted by centrifugation at 500 ***g*** for 5 min at 4 °C. Following separation by SDS-PAGE, proteins were transferred to a PVDF membrane and blocked in 10 % skimmed milk in PBS**/**0.1 % Tween (PBST). The membrane was incubated with primary antibody in 5 % skimmed milk in PBST, washed in PBST and then incubated with HRP-conjugated secondary antibody and washed as for the primary antibody.

#### Virus replication and release assay.

Huh7 cells were washed twice in DEPC-treated PBS before electroporating 2×10^6^ cells in DEPC-PBS with 1 µg RNA at 950 µF and 270 V. The cells were resuspended in complete medium and seeded at 1×10^6^ cells per well into six-well plates. At 72 h p.e., the cells were split at 1 : 5 before incubating for a further 72 h. At 144 h p.e., the cells were harvested in 400 µl TRIzol for qRT-PCR analysis, whilst the supernatants were removed and clarified at 2800 ***g*** for 5 min at room temperature before storing at −80 °C.

#### Virus titre by focus-forming assay.

Virus supernatants were clarified at 2800 ***g*** for 5 min at room temperature before being titrated on Huh7 cells as follows. In the 96-well format, clarified virus supernatants were serially diluted fivefold in complete DMEM plus HEPES before the addition of 100 µl diluted virus to Huh7 cells seeded 24 h previously into 96-well plates at 8×10^3^ cells per well (final volume 200 µl). The cells were incubated under normal cell-culture conditions for 72 h before washing in PBS and fixing in 4 % paraformaldehyde for 20 min. The cells were permeabilized in 0.1 % Triton X-100, 10 % FBS in PBS for 7 min followed by staining with anti-NS5A serum (Macdonald *et al.*, 2003) diluted 1 : 5000, followed by a corresponding secondary fluorescent antibody. The foci were counted manually to determine virus titres.

#### RNA extraction and qRT-PCR.

To quantify the number of HCV genomes, total cell RNA was extracted using TRIzol following manufacturer’s instructions (Invitrogen). Total extracted cellular RNA (100 ng) was analysed by qRT-PCR using a one-step qRT-PCR Taqman-based kit as directed by the manufacturer (Eurogentec). The primers and probe designed against the 5′-UTR have been described previously (Takeuchi *et al.*, 1999).

#### CsA treatment, EC_50_ and CC_50_.

Huh7 cells were electroporated and seeded as described for the luciferase-based replication assay. At 4 h p.e., the cells were treated with CsA at concentrations ranging from 0.01 to 100 µM (0.5 % DMSO final), incubated under normal conditions and harvested at 48 h p.e. in 30 µl Passive Lysis Buffer, and the luciferase activity was determined as described above. The data were modelled and the EC_50_ was calculated using Prism (GraphPad). Cytotoxicity assays were carried out in parallel to determine the CC_50_ using a MTT-based system (Sigma Aldridge).

#### Statistics.

Data generated from the luciferase assay and the virus replication/release assays were subjected to Student’s *t*-test assuming a one-tailed, unequal variance to determine statistical difference from the WT.

## References

[r1] Appel N. Pietschmann T. Bartenschlager R. **(**2005**).** Mutational analysis of hepatitis C virus nonstructural protein 5A: potential role of differential phosphorylation in RNA replication and identification of a genetically flexible domain. J Virol 79, 3187–3194 10.1128/JVI.79.5.3187-3194.2005 15709040 PMC548472

[r2] Appel N. Zayas M. Miller S. Krijnse-Locker J. Schaller T. Friebe P. Kallis S. Engel U. Bartenschlager R. **(**2008**).** Essential role of domain III of nonstructural protein 5A for hepatitis C virus infectious particle assembly. PLoS Pathog 4, e1000035 10.1371/journal.ppat.1000035 18369481 PMC2268006

[r3] Chatterji U. Lim P. Bobardt M. D. Wieland S. Cordek D. G. Vuagniaux G. Chisari F. Cameron C. E. Targett-Adams P. **& other authors (**2010**).** HCV resistance to cyclosporin A does not correlate with a resistance of the NS5A-cyclophilin A interaction to cyclophilin inhibitors. J Hepatol 53, 50–56 10.1016/j.jhep.2010.01.041 20451281 PMC2884070

[r4] Coelmont L. Hanoulle X. Chatterji U. Berger C. Snoeck J. Bobardt M. Lim P. Vliegen I. Paeshuyse J. **& other authors (**2010**).** DEB025 (Alisporivir) inhibits hepatitis C virus replication by preventing a cyclophilin A induced *cis*-*trans* isomerisation in domain II of NS5A. PLoS ONE 5, e13687 10.1371/journal.pone.0013687 21060866 PMC2965138

[r5] Evans M. J. Rice C. M. Goff S. P. **(**2004**).** Phosphorylation of hepatitis C virus nonstructural protein 5A modulates its protein interactions and viral RNA replication. Proc Natl Acad Sci U S A 101, 13038–13043 10.1073/pnas.0405152101 15326295 PMC516513

[r6] Feuerstein S. Solyom Z. Aladag A. Favier A. Schwarten M. Hoffmann S. Willbold D. Brutscher B. **(**2012**).** Transient structure and SH3 interaction sites in an intrinsically disordered fragment of the hepatitis C virus protein NS5A. J Mol Biol 420, 310–323 10.1016/j.jmb.2012.04.023 22543239

[r7] Fischer G. Gallay P. Hopkins S. **(**2010**).** Cyclophilin inhibitors for the treatment of HCV infection. Curr Opin Investig Drugs 11, 911–918 20721833

[r8] Foster T. L. Belyaeva T. Stonehouse N. J. Pearson A. R. Harris M. **(**2010**).** All three domains of the hepatitis C virus nonstructural NS5A protein contribute to RNA binding. J Virol 84, 9267–9277 10.1128/JVI.00616-10 20592076 PMC2937630

[r9] Foster T. L. Gallay P. Stonehouse N. J. Harris M. **(**2011**).** Cyclophilin A interacts with domain II of hepatitis C virus NS5A and stimulates RNA binding in an isomerase-dependent manner. J Virol 85, 7460–7464 10.1128/JVI.00393-11 21593166 PMC3126559

[r10] Fridell R. A. Qiu D. Wang C. Valera L. Gao M. **(**2010**).** Resistance analysis of the hepatitis C virus NS5A inhibitor BMS-790052 in an *in vitro* replicon system. Antimicrob Agents Chemother 54, 3641–3650 10.1128/AAC.00556-10 20585111 PMC2935007

[r11] Gao M. Nettles R. E. Belema M. Snyder L. B. Nguyen V. N. Fridell R. A. Serrano-Wu M. H. Langley D. R. Sun J.-H. **& other authors (**2010**).** Chemical genetics strategy identifies an HCV NS5A inhibitor with a potent clinical effect. Nature 465, 96–100 10.1038/nature08960 20410884 PMC7094952

[r12] Goh P.-Y. Tan Y.-J. Lim S. P. Lim S. G. Tan Y. H. Hong W. J. **(**2001**).** The hepatitis C virus core protein interacts with NS5A and activates its caspase-mediated proteolytic cleavage. Virology 290, 224–236 10.1006/viro.2001.1195 11883187

[r13] Griffin S. **(**2010**).** Inhibition of HCV p7 as a therapeutic target. Curr Opin Investig Drugs 11, 175–181 20112167

[r14] Hanoulle X. Badillo A. Wieruszeski J. M. Verdegem D. Landrieu I. Bartenschlager R. Penin F. Lippens G. **(**2009a**).** Hepatitis C virus NS5A protein is a substrate for the peptidyl-prolyl *cis*/*trans* isomerase activity of cyclophilins A and B. J Biol Chem 284, 13589–13601 10.1074/jbc.M809244200 19297321 PMC2679460

[r15] Hanoulle X. Verdegem D. Badillo A. Wieruszeski J. M. Penin F. Lippens G. **(**2009b**).** Domain 3 of non-structural protein 5A from hepatitis C virus is natively unfolded. Biochem Biophys Res Commun 381, 634–638 10.1016/j.bbrc.2009.02.108 19249289

[r16] He Y. Staschke K. A. Tan S.-L. **(**2006**).** HCV NS5A: a multifunctional regulator of cellular pathways and virus replication. In Hepatitis C Viruses: Genomes and Molecular Biology, pp. 267–292 Edited by Tan S. L. Norfolk, UK: Horizon Bioscience 21250384

[r17] Hughes M. Griffin S. Harris M. **(**2009a**).** Domain III of NS5A contributes to both RNA replication and assembly of hepatitis C virus particles. J Gen Virol 90, 1329–1334 10.1099/vir.0.009332-0 19264615 PMC7615708

[r18] Hughes M. Gretton S. Shelton H. Brown D. D. McCormick C. J. Angus A. G. Patel A. H. Griffin S. Harris M. **(**2009b**).** A conserved proline between domains II and III of hepatitis C virus NS5A influences both RNA replication and virus assembly. J Virol 83, 10788–10796 10.1128/JVI.02406-08 19656877 PMC2753128

[r19] Hwang J. Huang L. Cordek D. G. Vaughan R. Reynolds S. L. Kihara G. Raney K. D. Kao C. C. Cameron C. E. **(**2010**).** Hepatitis C virus nonstructural protein 5A: biochemical characterization of a novel structural class of RNA-binding proteins. J Virol 84, 12480–12491 10.1128/JVI.01319-10 20926572 PMC3004340

[r20] Jirasko V. Montserret R. Appel N. Janvier A. Eustachi L. Brohm C. Steinmann E. Pietschmann T. Penin F. Bartenschlager R. **(**2008**).** Structural and functional characterization of nonstructural protein 2 for its role in hepatitis C virus assembly. J Biol Chem 283, 28546–28562 10.1074/jbc.M803981200 18644781 PMC2661407

[r21] Jones D. M. Patel A. H. Targett-Adams P. McLauchlan J. **(**2009**).** The hepatitis C virus NS4B protein can *trans*-complement viral RNA replication and modulates production of infectious virus. J Virol 83, 2163–2177 10.1128/JVI.01885-08 19073716 PMC2643717

[r22] Liang Y. Ye H. Kang C. B. Yoon H. S. **(**2007**).** Domain 2 of nonstructural protein 5A (NS5A) of hepatitis C virus is natively unfolded. Biochemistry 46, 11550–11558 10.1021/bi700776e 17880107

[r23] Love R. A. Brodsky O. Hickey M. J. Wells P. A. Cronin C. N. **(**2009**).** Crystal structure of a novel dimeric form of NS5A domain I protein from hepatitis C virus. J Virol 83, 4395–4403 10.1128/JVI.02352-08 19244328 PMC2668466

[r24] Ma Y. Anantpadma M. Timpe J. M. Shanmugam S. Singh S. M. Lemon S. M. Yi M. **(**2011**).** Hepatitis C virus NS2 protein serves as a scaffold for virus assembly by interacting with both structural and nonstructural proteins. J Virol 85, 86–97 10.1128/JVI.01070-10 20962101 PMC3014171

[r25] Macdonald A. Harris M. **(**2004**).** Hepatitis C virus NS5A: tales of a promiscuous protein. J Gen Virol 85, 2485–2502 10.1099/vir.0.80204-0 15302943

[r26] Macdonald A. Crowder K. Street A. McCormick C. Saksela K. Harris M. **(**2003**).** The hepatitis C virus non-structural NS5A protein inhibits activating protein-1 function by perturbing Ras–ERK pathway signaling. J Biol Chem 278, 17775–17784 10.1074/jbc.M210900200 12621033

[r27] Moradpour D. Penin F. Rice C. M. **(**2007**).** Replication of hepatitis C virus. Nat Rev Microbiol 5, 453–463 10.1038/nrmicro1645 17487147

[r28] Shepard C. W. Finelli L. Alter M. J. **(**2005**).** Global epidemiology of hepatitis C virus infection. Lancet Infect Dis 5, 558–567 10.1016/S1473-3099(05)70216-4 16122679

[r29] Shimakami T. Hijikata M. Luo H. Ma Y. Y. Kaneko S. Shimotohno K. Murakami S. **(**2004**).** Effect of interaction between hepatitis C virus NS5A and NS5B on hepatitis C virus RNA replication with the hepatitis C virus replicon. J Virol 78, 2738–2748 10.1128/JVI.78.6.2738-2748.2004 14990694 PMC353754

[r30] Shimakami T. Yamane D. Jangra R. K. Kempf B. J. Spaniel C. Barton D. J. Lemon S. M. **(**2012**).** Stabilization of hepatitis C virus RNA by an Ago2–miR-122 complex. Proc Natl Acad Sci U S A 109, 941–946 10.1073/pnas.1112263109 22215596 PMC3271899

[r31] Shirota Y. Luo H. Qin W. Kaneko S. Yamashita T. Kobayashi K. Murakami S. **(**2002**).** Hepatitis C virus (HCV) NS5A binds RNA-dependent RNA polymerase (RdRP) NS5B and modulates RNA-dependent RNA polymerase activity. J Biol Chem 277, 11149–11155 10.1074/jbc.M111392200 11801599

[r32] Simmonds P. Bukh J. Combet C. Deléage G. Enomoto N. Feinstone S. Halfon P. Inchauspé G. Kuiken C. **& other authors (**2005**).** Consensus proposals for a unified system of nomenclature of hepatitis C virus genotypes. Hepatology 42, 962–973 10.1002/hep.20819 16149085

[r33] Takeuchi T. Katsume A. Tanaka T. Abe A. Inoue K. Tsukiyama-Kohara K. Kawaguchi R. Tanaka S. Kohara M. **(**1999**).** Real-time detection system for quantification of hepatitis C virus genome. Gastroenterology 116, 636–642 10.1016/S0016-5085(99)70185-X 10029622

[r34] Targett-Adams P. McLauchlan J. **(**2005**).** Development and characterization of a transient-replication assay for the genotype 2a hepatitis C virus subgenomic replicon. J Gen Virol 86, 3075–3080 10.1099/vir.0.81334-0 16227230

[r35] Tellinghuisen T. L. Marcotrigiano J. Rice C. M. **(**2005**).** Structure of the zinc-binding domain of an essential component of the hepatitis C virus replicase. Nature 435, 374–379 10.1038/nature03580 15902263 PMC1440517

[r36] Tellinghuisen T. L. Foss K. L. Treadaway J. **(**2008a**).** Regulation of hepatitis C virion production via phosphorylation of the NS5A protein. PLoS Pathog 4, e1000032 10.1371/journal.ppat.1000032 18369478 PMC2265800

[r37] Tellinghuisen T. L. Foss K. L. Treadaway J. C. Rice C. M. **(**2008b**).** Identification of residues required for RNA replication in domains II and III of the hepatitis C virus NS5A protein. J Virol 82, 1073–1083 10.1128/JVI.00328-07 18032500 PMC2224455

[r38] Wakita T. Pietschmann T. Kato T. Date T. Miyamoto M. Zhao Z. Murthy K. Habermann A. Kräusslich H. G. **& other authors (**2005**).** Production of infectious hepatitis C virus in tissue culture from a cloned viral genome. Nat Med 11, 791–796 10.1038/nm1268 15951748 PMC2918402

[r39] Yang F. Robotham J. M. Grise H. Frausto S. Madan V. Zayas M. Bartenschlager R. Robinson M. Greenstein A. E. **& other authors (**2010**).** A major determinant of cyclophilin dependence and cyclosporine susceptibility of hepatitis C virus identified by a genetic approach. PLoS Pathog 6, e1001118 10.1371/journal.ppat.1001118 20886100 PMC2944805

[r40] Yang P. L. Gao M. Lin K. Liu Q. Villareal V. A. **(**2011**).** Anti-HCV drugs in the pipeline. Curr Opin Virol 1, 607–616 10.1016/j.coviro.2011.10.019 22440918 PMC3775341

